# Microbial Enzyme: Applications in Industry and in Bioremediation

**DOI:** 10.1155/2012/980681

**Published:** 2012-01-05

**Authors:** Alane Beatriz Vermelho, Claudiu T. Supuran, Jose M. Guisan

**Affiliations:** ^1^Departamento de Microbiologia Geral, Instituto de Microbiologia Paulo de Góes, 21949-900 Universidade Federal do Rio de Janeiro-UFRJ CCS, Bloco I, Cidade Universitária, Ilha do Fundão, Rio de Janeiro, RJ, Brazil; ^2^Chim Bioorgan Lab, Università degli Studi di Firenze, 50019 Florence, Italy; ^3^Departamento de Biocatálisis, Instituto de Catálisis, CSIC, Campus UAM, Cantoblanco, 28049 Madrid, Spain

This special issue is dedicated to the study of microbial enzymes and their applications in various industries. The biocatalytic uses for enzymes have grown immensely in recent years since they are ecologically correct, have a high specificity, present chemo-regio-enantio selectivity, and have a wide diversity of reactions. Moreover, the conditions to obtain and optimize the production of enzymes in terms of nutrients, pH, temperature, and aeration are easily controlled in bioreactors. Microorganisms can also be manipulated genetically to improve the desirable characteristics of a biocatalyzer. Additionally, the substrates used in the cultural medium are sustainable and industrial residuals can be used to produce value-added products. All these characteristics together have encouraged the ever-growing search for biocatalytic processes. The main industries that apply microbial enzymes are the food, textile, leather, pharmaceutical, cosmetics, fine chemicals, energy, biomaterials, paper, cellulose and detergent industries. Immobilization processes allow the reuse of these enzymes and increase stability. The enzymes and the microorganisms themselves have also been much used for bioremediation processes. 

In this issue, the paper by B. Joseph et al. is about the production of cold-active lipases by semisolid fermentation and the paper by A. L. Willerding et al. is a study about lipases obtained from microorganisms isolated from soils in the Amazon. Studies with lipases immobilized for the synthesis of isopropyl acetate and isopropyl ferulate are presented in the papers by M. Lal Verma et al., A. Kumar and S. S. Kanwar, respectively.

The results of cellulase production optimization studies with cellulosic substrates and delignified *Bambusa bambos* are presented in the papers by D. Deka et al. and A. Kuila et al. The paper by R. C. Kuhad et al. is a review of the innumerous industrial applications of the cellulases.

 The papers entitled *“Bioconversion of agricultural waste to ethanol by SSF using recombinant cellulase from clostridium thermocellum,” “Petroleum-degrading enzymes: bioremediation and new prospects,” *and *“Assessment of the morphological, biochemical, and kinetic properties for Candida rugosa lipase*

*immobilized on hydrous niobium oxide to be used in the biodiesel synthesis”* are about enzymes and biofuels: the use of a recombinant cellulase from *Clostridium thermocellum *for the production of ethanol from agro-industrial residues, a brief review on the role of enzymes that degrade oil, the use and description of the properties of a *Candida rugosa* lipase immobilized for the production of biodiesel.

 The paper entitled “*Laccase: microbial sources, production, purification, and potential biotechnological applications”* is a review of the industrial applications of laccases, while the “*Isolation, purification, and characterization of fungal laccase from Pleurotus sp.”* and “*Application of asymetrical and Hoke designs for optimization of laccase production by the white-rot fungus Fomes fomentarius in solid-state fermentation*” are studies on laccases from fungi and production optimization. The paper by A. Arsenault et al. shows that a laccase from *Coriolopsis polyzona* was insolubilized as cross-linked enzyme aggregates (CLEAs) for the first time with chitosan as the cross-linking agent. Also, within the laccase theme, the paper entitled “*VoEnzyme-catalyzed oxidation of 17*β*-estradiol using immobilized laccase from Trametes versicolor*” is about the oxidation of 17*β*-estradiol using immobilized laccase from *Trametes versicolor. *The paper by M. Neifar et al. is about the decolorization of Solophenyl red 3BL (SR), a polyazo dye extensively used in the textile industry using a laccase-mediator system. The last article about laccase, entitled “*Improved laccase production by Trametes pubescens MB 89 in distillery wastewaters,*” is a study of its production by *Trametes pubescens* using distillery wastewaters.

The paper by A. S. Galdino et al. describes the characterization of amylases of the *Cryptococcus flavus* fungus expressed in the yeast *Saccharomyces cerevisiae. *The paper by I. Akpan et al. demonstrated the use of activated Charcoal in order to recovery glucoamylase. 

 The paper by E. F. Ries and G. A. Macedo reports on the improvement of phytase activity of a new strain of *S. cerevisiae* using optimization statistics.

 The paper by A. Flores-Maltos et al. describes the catalytical properties of free and immobilized *Aspergillus niger* tannase, and, in the paper by L. V. Rodríguez-Durán et al., novel strategies for upstream and downstream processing of tannin acyl hydrolase are described.

The paper by M. F. S. Teixeira et al. shows the improvements that occur in cupuacu juice after treatment with crude enzyme extract produced by *Aspergillus japonicus* 586 in the food industry. The use of proteases with potential in the food and animal feed industry are focused on in two articles: the one by B. Tchorbanov et al. describes the use of *Lactobacillus* LBL-4 proteases to remove the bitter taste of proteinic hydrolysates, and the paper by A. M. Mazotto et al. describes how strains of *Bacillus* sp. were used to ferment feather flour so that it could be transformed into more easily absorbed hydrolysates. And within the same theme, the paper by F. C. Lopes et al. shows that a strain of *Aspergillus niger* was able to grow in feather meal producing proteases and keratinases.

Optimization of the proteases from *Bacillus licheniformis *NCIM 2042 and the effect of different culture media on *Bacillus* sp. isolated from soil sample of Lavizan Jungle Park are focused on in the papers by B. Bhunia and A. Dey; L. Jabalameli and A. A. Sepahy, respectively.

The xylanase enzymes are described in the paper by A. A. Sepahy et al. that reports on the cost effective production and optimization of this enzyme (alkaline xylanase) using indigenous *Bacillus mojavensis* AG137 with agricultural residues. 

In the paper by K. Praveen et al., the lignolytic enzymes of the mushroom *Stereum ostrea,* isolated from wood logs, are described.

Naringinase (NGase) is an enzyme complex with high potential for the pharmaceutical and food industries. The paper by M. H. L. Ribeiro and M. Rabaça describes the process of enzyme immobilization with cross-linked enzyme aggregates (CLEAs). The enzymatic synthesis of the flavone glucosides, prunin and isoquercetin, and the aglycones, naringenin, and quercetin, with selective *α*-l-rhamnosidase and *β*-D-glucosidase activities of naringinase is described in the paper by H. Vila-Real et al.

In the paper by J. de A. Figueira et al., a set of supports (Eupergit, Amberlite, alginate, gelatin, polyvinyl alcohol-PVA-) based matrices (Lentikats), and sol-gel) for the immobilization of a partially purified extract of *β*-glucosidase was screened.

The paper by S. Ou et al. describes the production of feruloyl esterase from *Aspergillus niger* by solid-state fermentation using different substrates, and the paper by S. Shukla and A. Goyal identifies the hyperproductive strains of glucan.

We believe that this special issue that gathers together updated studies under the theme “*Microbial Enzyme: Applications in Industry and in Bioremediation*” offers an important contribution to microbial enzyme studies, encompassing their production and optimization, immobilization techniques and, industrial applications. We would like to thank the collaboration of all the authors and the reviewers for their analyses of the papers. 

*Alane Beatriz Vermelho* *Alane Beatriz Vermelho* *Claudiu T. Supuran* *Claudiu T. Supuran* *Jose M. Guisan* *Jose M. Guisan*

